# The Association Between Cultural Tightness and COVID-19 Vaccine Confidence From 28 Countries: Cross-Sectional Study

**DOI:** 10.2196/66872

**Published:** 2025-04-24

**Authors:** Qiang Wang, Ana Bolio, Leesa Lin

**Affiliations:** 1 Laboratory of Data Discovery for Health (D24H), Hong Kong Science Park, Hong Kong Special Administrative Region, China (Hong Kong); 2 Department of Infectious Disease Epidemiology, London School of Hygiene & Tropical Medicine, Keppel Street, London, WC1E 7HT, United Kingdom, +852 3910 3770; 3 WHO Collaborating Centre for Infectious Disease Epidemiology and Control, School of Public Health, Li Ka Shing Faculty of Medicine, The University of Hong Kong, Hong Kong Special Administrative Region, China (Hong Kong)

**Keywords:** COVID-19, vaccine confidence, social norms, global, cultural tightness

## Abstract

**Background:**

Social norms provided a framework for understanding a variety of behaviors. Cultural tightness was introduced to measure the level of adherence to social norms and tolerance of deviant behavior.

**Objective:**

We aimed to explore the association between cultural tightness and COVID-19 vaccine hesitancy.

**Methods:**

A total of 44,339 participants aged 18 years and older were enrolled from 28 different countries between 2020 and 2022. We used the Vaccine Confidence Index (3 items related to evaluation of importance, effectiveness, and safety) with a 5-point Likert scale to collect COVID-19 vaccine confidence. Demographic information at the individual-level was obtained through the survey, while national-level data were sourced from the World Bank and Hofstede insights. Multilevel linear regressions with random effects for country were used to examine the association between cultural tightness and COVID-19 vaccine confidence.

**Results:**

Of the participants, 21,968 (50.2%) were male and 18,957 (43.3%) had an education level of university or above. Vietnam exhibited the highest level of confidence (mean 13.31, SD 1.71) on COVID-19 vaccine and Slovakia had the lowest level (mean 9.52, SD 0.14). The higher levels of cultural tightness were positively linked to greater vaccine confidence (β=1.94, 95% CI 1.72-2.15; *P*<.001) after controlling individual- and national- level variables. Individuals who were younger in age, female, had lower levels of educational level, or belonged to minority religious groups demonstrated a positive association with lower vaccine confidence. Hofstede’s 5 cultural dimensions were not significantly associated with vaccine confidence. The level of vaccine confidence in 2021 (β=−0.54, 95% CI −0.67 to −0.37; *P*<.001) and 2022 (β=−0.23, 95% CI −0.34 to −0.10; *P*<.001) was lower than that observed in 2020.

**Conclusions:**

Lower level of cultural tightness might be positively associated with low vaccine confidence. Our findings offered the insight for designing tailor interventions to vaccine hesitancy in different cultural tightness context.

## Introduction

According to the World Health Organization (WHO), vaccine hesitancy refers to the “delay in acceptance or refusal of vaccination despite availability of vaccination services” [1], which could lead to a decrease in vaccination uptake and the re-emergence of vaccine-preventable diseases, such as measles [2]. The WHO has identified vaccine hesitancy as one of the ten greatest threats to global health [3], and COVID-19 vaccine hesitancy has attracted a lot of attention [4,5].

The acceptance of COVID-19 vaccination varied across countries: among 23 countries in a global survey, the COVID-19 vaccine acceptance rate ranged between 51.6% (Russia) and 97.6% (China) [5]. Much scholarly effort has been directed toward exploring the driving factors behind COVID-19 vaccine hesitancy [5,6], which often focused on the scale of individual-level factors, such as sociodemographics, that could not fully explain the wide variations observed among countries. Some researchers found that countries with higher levels of trust in government [4] and science [7] tended to have higher COVID-19 vaccine acceptance. According to Lin et al [8], there was a negative association between COVID-19 vaccine confidence and a higher misinformation belief level across 14 countries.

Social norms are characterized as beliefs that are collectively shared regarding what is considered typical or concerning what is expected behavior within a group, which guide individuals’ behaviors. It provided a framework for understanding a variety of behaviors, including encouraging energy conservation [9], charitable donations [10], and health decisions [11]. Cultural tightness was introduced by Gelfand et al [12] to measure the degree of adherence to norms within a country (or a state within a country) as well as the level of tolerance for individuals who deviate from these norms. The societies characterized by strong norms and a low tolerance of deviant behavior could be classified as exhibiting high cultural tightness, whereas those with weak norms and a high tolerance of deviant behavior could be classified as exhibiting low cultural tightness [12].

The high ecological validity of cultural tightness has been observed across a variety of domains, including politics (leadership preference), economics (stock price synchrony), and culture (prejudice) [13]. The cultural tightness was also associated with countries’ response to the pandemic. Countries with high cultural tightness were more likely to show lower number of COVID-19 cases and COVID-19–related mortality rates than countries with low cultural tightness [13]. The association between vaccine confidence and cultural tightness across countries has been examined. A previous study found a negative correlation between cultural tightness and willingness to receive COVID-19 vaccination [14]. In a study conducted by Lu [15], cultural tightness did not significantly correlate with COVID-19 vaccine hesitancy across 22 countries. In a study by Shi et al [16], the positive correlation between cultural tightness and vaccination willingness became negative considering variables related to individual and collective norms in 8 Asian countries. The inconsistent findings urged the further studies.

This study aimed to examine the association between COVID-19 vaccine confidence and cultural tightness on a global scale. To achieve this goal, we conducted a multilevel analysis of 43,744 individuals from 28 countries, adjusting for numerous individual and national variables. The findings could help to understand causes of heterogeneity in vaccine confidence across countries and provide insights on developing tailored and effective interventions to deal with vaccine hesitancy.

## Methods

### Data

The Vaccine Confidence Project (VCP) is a research group dedicated to monitoring, analyzing, and mitigating public concerns regarding vaccine, which used a range of methods—including surveys, social listening, and artificial intelligence tool. The VCP has conducted multiple surveys about COVID-19 vaccine confidence covering more than 70 countries and territories with participants aged>18 years [17-19]. A total of 44,339 individuals were included from 28 countries, which provided the available data about cultural tightness and COVID-19 vaccine confidence. The sample sizes varied across the countries surveyed, with the smallest sample being 1000 participants (in Czechia, Estonia, Finland, Greece, Hungary, Netherlands, Poland, Portugal, Slovak Republic, Spain, Sweden, United Kingdom, Ecuador, Peru, and Brazil) and the largest being 4358 participants (in Nigeria). The mean sample size across all countries was 1584 individuals (Figure S1 in Multimedia Appendix 1). A total of 24 countries were surveyed in 2020, 4 in 2021, and 6 in 2022. We provided the detailed field date in Table S1 in Multimedia Appendix 1. South Korea was surveyed in all 3 years, while Nigeria and Kenya were surveyed in 2020 and 2022, and Vietnam, Japan, and Malaysia were surveyed in 2021 and 2022. We performed the survey in collaboration with Opinion Research Business International [20]. Fieldwork was performed through face-to-face interviews, telephone interviews, and online. Samples were selected at random and stratified to align with the proportions of demographic distributions for age, sex, and subnational region in each country. Before participating in the surveys, all respondents provided informed consent.

### Exposure

We obtained national-level cultural tightness scores from a previous study [21]. This study, which included 22,863 participants across 57 countries, measured cultural tightness using a 6-item scale (Table S2 in Multimedia Appendix 1) [21]. The responses from surveyed individuals within each country were then analyzed to derive a national-level cultural tightness score. To avoid confusion between positive and negative descriptions in the original concept, we adopted the term “cultural tightness,” with higher values indicating tighter culture. Among the 28 countries evaluated, Hungary exhibited the lowest cultural tightness, while India demonstrated the highest level.

### Outcome

We used 3 items from the Vaccine Confidence Index (VCI) to evaluate participants’ level of confidence (COVID-19 vaccines are important, COVID-19 vaccines are effective, and COVID-19 vaccines are safe) [22]. Answers were measured on a 5-point Likert scale, ranging from “strongly agree=5 points” to “strongly disagree=1 point.” We added up the scores of the 3 items to construct a composite measurement of overall COVID-19 vaccine confidence. Furthermore, the surveyed items related to importance, effectiveness, and safety of vaccine were also analyzed as outcomes separately.

### Covariates

Participants’ sociodemographic variables were considered in the analysis, including age group (18‐24 y vs 25‐34 y vs 35‐44 y vs 45‐54 y vs ≥55 y), sex (male vs female), education level (primary or below vs secondary vs university or above vs other educational level), and religion (largest vs minority vs refusal to answer). Regarding religion, the most commonly reported religious beliefs within a country were classified as the “largest,” while all other religious beliefs were grouped as “minority.” Respondents who declined to answer were considered as “refusal to answer.” Individual perception towards vaccine was surveyed and analyzed as a potential confounder by the statement “Vaccines are important for people of all ages to have” on a 5-point Likert scale. We considered an interaction between individual perception and cultural tightness, given that the association between cultural tightness and vaccine confidence may vary according to different levels of individual perception. We adjusted for the potential impact of national-level factors by including gross national income (GNI) in 2021, converted by the Atlas method, population density (number of people per square km), and survey year (ie, 2020 vs 2021 vs 2022; see Figure S2 in Multimedia Appendix 1). These indicators were derived from the World Bank. Given the potential response biases caused by multiple data collection methods, the survey methodology was incorporated as a covariate in the regression model. In addition, based on a previous study [15], we controlled some cultural dimensions as defined by Hofstede [23]. The use of cultural dimensions theory has served as a valuable tool for comprehending cross-country cultural variations. We included individualism (indicating the degree of emphasis on individual interests over collective or group interests), power distance (reflecting the extent of acceptance and anticipation of unequal power and authority distribution among society’s members), long-term orientation (pertaining to the degree of emphasis on future-oriented values), uncertainty avoidance (the extent to which individuals attempt to cope with anxiety by minimizing uncertainty), and indulgence (pertaining to the extent of allowance for and enjoyment of basic human desires and pleasures). The motivation toward achievement and success dimension were excluded because theoretical considerations suggest it was less likely to significantly influence the association between cultural tightness and vaccine confidence.

### Statistical Analysis

The percentage of missing data for individual variables was below 0.9% across all countries (Table S3 in Multimedia Appendix 1). Since the percentage of missing data was low, cases with at least one missing variable were excluded from our analysis. Multilevel linear regressions with random effects for country (which accounts for the clustering within country) were used to examine the association between cultural tightness and COVID-19 vaccine confidence. Univariate analysis (model 1) was performed to examine the relationship between each individual- and-country-level factor and vaccine confidence with clustering by country. We controlled the individual-level variables in the model 2, and all individual- and-country-level variables in the model 3. Variance inflations factors (VIFs) were used to quantify the level of multicollinearity; the VIF for variables below 5 indicated there was only a moderate level of multicollinearity. All country-level variables were standardized in the model. We used 2-sided *P* values and statistical significance was set at *P*<.05. All analyses were performed using R software (R Foundation for Statistical Computing).

### Ethical Considerations

The data collection across the 28 countries included in this study received ethics approval from the University of Hong Kong (EA230420) and the London School of Hygiene & Tropical Medicine (26636; 22130). All surveys were conducted in accordance with local regulations and institutional ethical requirements, and informed consent was obtained from participants at the time of data collection. For the current analysis, which used an aggregated and deidentified dataset, additional ethical approval was not required in accordance with VCP policies governing the use of anonymized secondary data. The original informed consents explicitly included provisions for future research use, including secondary analyses.

## Results

**Figure 1.**
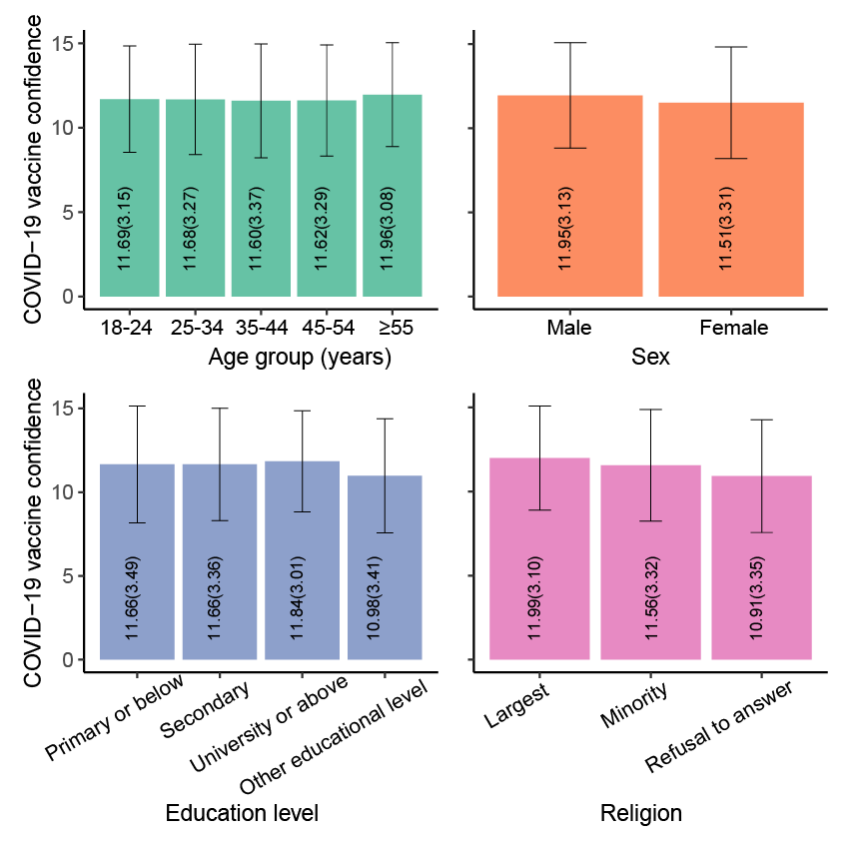
Mean COVID-19 vaccine confidence with SD by various sociodemographics among 43,744 individuals.

**Figure 2.**
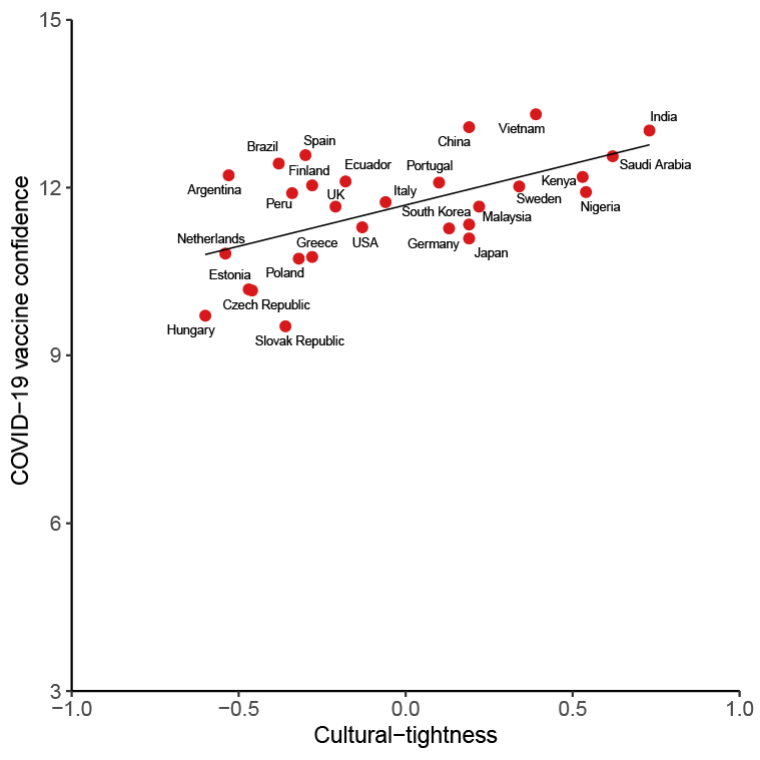
Scatter plot of cultural tightness and mean COVID-19 vaccine confidence. The x-axis shows the cultural tightness score and the y-axis represents the mean COVID-19 vaccine confidence. A fitted regression line was included to illustrate the trend without controls.

A total of 43,744 participants were included (Table 1). Of these participants, 21,968 (50.2%) were male and 4351 (9.9%) had an education level of primary education or below. The mean COVID-19 vaccine confidence was higher among males compared with females (mean 11.95, SD 3.13 vs mean 11.51, SD 3.31; see Figure 1). Vietnam had the highest mean vaccine confidence at 13.31 (SD 1.71), followed by China at 13.08 (1.71). Conversely, Slovakia had the lowest level at 9.52 (SD 0.14), followed by Hungary with the second-lowest level at 9.71 (SD 0.13). India had the largest proportions of individuals who strongly agreed with the 3 items regarding confidence (1004/1341, 74.9% on importance; 761/1341, 56.7% on safety; and 788/1341, 58.8% on effectiveness), and Hungary had the largest proportions of individuals who strongly disagreed with the 3 items (213/999, 21.3% on importance; 221/999, 22.1% on safety; and 277/999, 27.7% on effectiveness; Figures S3-S5 in Multimedia Appendix 1). Cultural tightness was positively associated with mean vaccine confidence (Spearman correlation coefficient, ρ=0.51; *P*=.005; n=28) without controls. Figure 2 illustrated the variation in vaccine confidence across different levels of cultural tightness.

Our study identified significant correlations between cultural tightness and COVID-19 vaccine confidence. In Table 2, we observed that higher levels of cultural tightness were positively linked to greater vaccine confidence (β=1.94, 95% CI 1.72-2.15; *P*<.001) after adjusting for individual and country-level variables in model 3. There was 1-unit increase in standardized cultural tightness being associated with an average increase of 1.94 units in vaccine confidence score. Individuals aged ≥55 years were more likely to have higher vaccine confidence compared with those aged 18‐24 years (β=0.35, 95% CI 0.27-0.43; *P*<.001). Female participants had lower vaccine confidence than male participants (β=−0.29, 95% CI −0.34 to −0.25; *P*<.001). Participants with an education level of university or above were more likely to have higher vaccine confidence than those with a primary education level or below (β=0.16, 95% CI 0.07-0.25; *P*<.001). Individuals with minority religious beliefs exhibited lower vaccine confidence (β=−0.13, 95% CI −0.18 to −0.08; *P*<.001) versus those with largest religious beliefs. The level of vaccine confidence in 2021 and 2022 was lower than that observed in 2020. All variables have VIFs lower than 5 (Table S4 in Multimedia Appendix 1).

Increased agreement on the importance, effectiveness, and safety of the COVID-19 vaccine was positively correlated with a higher level of cultural tightness (Table 3). Female participants demonstrated a lower likelihood of agreeing on these 3 items compared with male participants. A positive association was identified between a higher GNI and greater agreement on the importance of the COVID-19 vaccine (β=0.11, 95% CI 0.04-0.19; *P*=.03). Over time, there was a decreasing trend in agreements on the importance and effectiveness of the COVID-19 vaccine.

**Table 1.** Sample characteristics.

Variables	Results (N=43,744)
Age group (years), n (%)	
18‐24	6900 (15.8)
25‐34	9899 (22.6)
35‐44	8561 (19.6)
45‐54	7086 (16.2)
≥55	11,298 (25.8)
Sex, n (%)	
Male	21,968 (50.2)
Female	21,776 (49.8)
Education level, n (%)	
Primary or below	4351 (9.9)
Secondary	19,841 (45.4)
University or above	18,957 (43.3)
Other educational level	595 (1.4)
Religion, n (%)	
Largest	21,680 (49.6)
Minority	19,264 (44)
Refusal to answer	2800 (6.4)
Individual perceptions, mean (SE)	4.17 (1.14)

**Table 2.** Multilevel logistic regression analyses for associations of factors and COVID-19 vaccine confidence.

Variables	Model 1 (crude)		Model 2 (variables in the individual-level)		Model 3 (variables in the individual-and country-level)	
	β (95% CI)	*P* value	β (95% CI)	*P* value	β (95% CI)	*P* value
Cultural tightness	0.56 (0.26 to 0.85)	.001	2.13 (1.95 to 2.30)	<.001	1.94 (1.72 to 2.15)	<.001
Individual level						
Age group, years (18‐24 years as reference)						
25‐34	−0.06 (−0.16 to 0.04)	.22	−0.10 (−0.18 to −0.02)	.02	−0.10 (−0.18 to −0.02)	.02
35‐44	0.00 (−0.10 to 0.10)	.99	−0.05 (−0.13 to 0.03)	.24	−0.05 (−0.13 to 0.03)	.24
45‐54	0.16 (0.05 to 0.26)	.003	0.03 (−0.06 to 0.12)	.51	0.03 (−0.06 to 0.12)	.52
≥55	0.76 (0.66 to 0.86)	<.001	0.34 (0.26 to 0.42)	<.001	0.35 (0.27 to 0.43)	<.001
Sex (male as reference)						
Female	−0.39 (−0.45 to −0.33)	<.001	−0.29 (−0. 34 to −0.25)	<.001	−0.29 (−0.34 to −0.25)	<.001
Education level (primary or below as reference)						
Secondary	0.08 (−0.02 to 0.19)	.13	−0.03 (−0.12 to 0.06)	.49	−0.03 (−0.11 to 0.06)	.55
University or above	0.31 (0.20 to 0.41)	<.001	0.14 (0.05 to 0.23)	.002	0.16 (0.07 to 0.25)	<.001
Other educational level	−0.48 (−0.75 to −0.22)	<.001	−0.43 (−0.65 to −0.21)	<.001	−0.42 (−0.64 to −0.20)	<.001
Religion (largest as reference)						
Minority	0.34 (−0.40 to −0.27)	<.001	−0.14 (−0.20 to −0.09)	<.001	−0.13 (−0.19 to −0.08)	<.001
Refusal to answer	−0.84 (−0.97 to −0.71)	<.001	−0.24 (−0.35 to −0.13)	<.001	−0.24 (−0.35 to −0.14)	<.001
Individual perception	1.53 (1.50 to 1.55)	<.001	1.48 (1.46 to 1.50)	<.001	1.48 (1.46 to 1.50)	<.001
Individual perception and cultural tightness	—^a^	—	−0.42 (−0.44 to −0.39）	<.001	−0.42 (−0.44 to −0.40)	<.001
Country level						
Individualism	−0.43 (−0.77 to −0.08)	.02	—	—	−0.28 (−0.52 to −0.04)	.08
Indulgence	0.02 (−0.39 to 0.44)	.92	—	—	0.046 (−0.23 to 0.14)	.70
Long-term orientation	−0.39 (−0.78 to 0.01)	.07	—	—	0.03 (−0.23 to 0.29)	.84
Power distance	0.16 (−0.20 to 0.52)	.40	—	—	0.00 (−0.21 to 0.21)	.98
Uncertainty avoidance	−0.28 (−0.62 to 0.06)	.12	—	—	−0.17 (−0.36 to −0.01)	.14
GNI^b^	0.16 (−0.28 to 0.59)	.49	—	—	0.19 (−0.01 to 0.39)	.13
Population density	0.02 (−0.36 to 0.40)	.91	—	—	0.13 (−0.07 to 0.32)	.30
Survey time (2020 as reference)						
2021	−0.65 (−0.82 to −0.48)	<.001	—	—	−0.54 (−0.67 to −0.37)	<.001
2022	−0.45 (−0.58 to −0.33)	<.001	—	—	−0.23 (−0.34 to −0.10)	<.001
Survey methodology (online as reference)						
CATI^c^	0.77 (0.08 to 1.47)	.04	—	—	0.11 (−0.31 to 0.53)	.69
Face-to-face	1.03 (0.32 to 1.74)	.009	—	—	0.58 (0.12 to 1.02)	.04

a Not available.

b GNI: gross national income.

c CATI: computer-assisted telephone interview.

**Table 3.** Multilevel logistic regression analyses for associations of factors and attitudes toward COVID-19 vaccine importance, effectiveness, and safety controlling individual-and-country level variables.

Variables	Importance		Effectiveness		Safety	
	β (95% CI)	*P* value	β (95% CI)	*P* value	β (95% CI)	*P* value
Cultural tightness	0.68 (0.60 to 0.76)	<.001	0.62 (0.54 to 0.69)	<.001	0.64 (0.55 to 0.73)	<.001
Individual level						
Age group, years (18‐24 years as reference)						
25‐34	−0.04 (−0.07 to −0.01)	.004	−0.04 (−0.07 to −0.01)	.22	−0.01 (−0.05 to 0.02)	.39
35‐44	−0.02 (−0.05 to 0.01)	.18	−0.03 (−0.07 to 0.00)	.06	0.005 (−0.03 to 0.04)	.78
45‐54	0.02 (−0.01 to 0.05)	.19	−0.01 (−0.04 to 0.03)	.76	0.012 (−0.02 to 0.05)	.50
≥55	0.15 (0.11 to 0.18)	<.001	0.10 (0.07 to 0.13)	<.001	0.10 (0.07 to 0.13)	<.001
Sex (male as reference)						
Female	−0.06 (−0.08 to −0.04)	<.001	−0.11 (−0.13 to −0.09)	<.001	−0.12 (−0.14 to −0.10)	<.001
Education level (primary or below as reference)						
Secondary	0.02 (−0.01 to 0.05)	.27	−0.04 (−0.08 to −0.00)	.03	−0.004 (−0.04 to 0.03)	.83
University or above	0.10 (0.07 to 0.13)	<.001	0.03 (−0.01 to 0.07)	.12	0.03 (−0.01 to 0.07)	.09
Other educational level	−0.08 (−0.17 to 0.00)	.06	−0.14 (−0.23 to −0.05)	.002	−0.20 (−0.29 to −0.11)	<.001
Religion (largest as reference)						
Minority	−0.04 (−0.06 to −0.02)	<.001	−0.05 (−0.07 to −0.03)	<.001	−0.04 (−0.07 to −0.02)	<.001
Refusal to answer	−0.10 (−0.14 to −0.06)	<.001	−0.06 (−0.10 to −0.02)	.01	−0.09 (−0.13 to −0.04)	<.001
Individual perception	0.51 (0.50 to 0.52)	<.001	0.47 (0.46 to 0.48)	<.001	0.50 (0.49 to 0.51)	<.001
Individual perception × cultural tightness	−0.15 (−0.17 to −0.14)	<.001	−0.13 (−0.14 to −0.12)	<.001	−0.14 (−0.15 to −0.13)	<.001
Country level						
Individualism	−0.15 (−0.24 to −0.06)	.02	−0.09 (−0.18 to −0.01)	.08	−0.04 (−0.13 to 0.06)	.57
Indulgence	−0.02 (−0.09 to 0.05)	.63	0.00 (−0.06 to 0.06)	.96	−0.03 (−0.10 to 0.05)	.60
Long-term orientation	0.01 (−0.09 to 0.10)	.93	0.03 (−0.06 to 0.12)	.57	−0.01 (−0.11 to 0.10)	.93
Power distance	−0.04 (−0.12 to 0.05)	.50	0.03 (−0.05 to 0.10)	.54	0.01 (−0.08 to 0.10)	.86
Uncertainty avoidance	−0.06 (−0.13 to 0.01)	.19	−0.06 (−0.12 to 0.00)	.15	−0.06 (−0.13 to 0.02)	.23
GNI^a^	0.11 (0.04 to 0.19)	.03	0.08 (0.01 to 0.14)	.09	0.00 (−0.08 to 0.08)	.95
Population density	0.07 (−0.01 to 0.14)	.18	0.04 (−0.02 to 0.11)	.30	0.02 (−0.06 to 0.10)	.70
Survey time (2020 as reference)						
2021	−0.34 (−0.38 to −0.27)	<.001	−0.11 (−0.16 to −0.04)	<.001	−0.09 (−0.15 to −0.03)	.004
2022	−0.25 (−0.29 to −0.19)	<.001	−0.01 (−0.05 to 0.05)	.70	0.02 (−0.02 to 0.08)	.33
Survey methodology (online as reference)						
CATI^b^	0.06 (−0.10 to 0.22)	.54	0.10 (−0.04 to 0.25)	.27	−0.06 (−0.23 to 0.12)	.60
Face-to-face	0.24 (0.05 to 0.40)	.04	0.16 (−0.00 to 0.32)	.10	0.18 (−0.01 to 0.36)	.12

a GNI: gross national income.

b CATI: computer-assisted telephone interview.

## Discussion

### Principal Findings

Our study found a significant association between cultural tightness and COVID-19 vaccine confidence. Countries with higher cultural tightness tended to have greater confidence in the vaccine, which may account for the disparities in vaccine confidence observed among countries worldwide. This finding not only facilitated progress in explaining vaccine hesitancy determinants from a cultural perspective, but also underscored the importance of developing tailored and effective interventions to address the issue.

In societies characterized by high cultural tightness, individuals were expected to adhere to social norms, minimize risks, and prioritize stability [24]. During the COVID-19 pandemic, individuals in these cultures demonstrated a greater tendency toward collective behavior and were more likely to strictly adhere to guidelines issued by authorities, including recommendations for vaccination [13,25]. Notably, individuals in high-tightness cultures would concern with the social consequences of norm deviation—such as the risk of social exclusion due to vaccine refusal—rather than solely the health risks associated with nonvaccination [12]. Furthermore, high-tightness cultures typically exhibited a centralized information environment dominated by official sources, which might serve to mitigate the spread of vaccine-related misinformation. Conversely, low cultural tightness societies were characterized by a more pluralistic information landscape, allowing for the sharing of diverse opinions on vaccination, which might increase the misinformation spread that reduced vaccine willingness [26]. In fact, historical evidence showed that the antivaccine movement first emerged in countries with loose cultures; for example, an image linking smallpox vaccination to cattle traits was published in a British magazine in the 18th century [27]. The movement against smallpox vaccination also first occurred in North America in the 19th century [28]. The antivaccination movement reemerged in the United Kingdom in the mid-1970s due to a report suggesting that the diphtheria, pertussis, and tetanus vaccine could lead to severe neurological diseases [28].

Our findings were inconsistent with the previous studies. Differences in analytical methods, the number of countries included, and the measurement items of vaccine confidence may account for the discrepancies between our results and those reported in earlier researches [14-16]. Ng and Tan [14] examined the association between cultural tightness and vaccination willingness across 12 countries without controlling for potential confounders, which limited the reliability of their results. They thought that negative association may be due to individuals in countries with higher culture tightness feeling a low perceived risk of COVID-19 infection due to effective control of the pandemic, resulting in decreased motivation to receive vaccinations [14]. Shi et al [16] focused primarily on East and Southeast Asian countries, where the cultural tightness scores measured were relatively high (more than half of the countries) and exhibited limited variability among 8 countries. Lu [15] investigated the associations in 22 countries. Furthermore, previous studies often relied on a single-item measure to assess vaccine confidence. Our study examined the association by employing VCI items, adjusting confounders, and including a larger, more diverse sample of countries.

Our study demonstrated that younger age, female sex, and low educational level were positively associated with low vaccine confidence, in line with findings from a systematic review of factors influencing COVID-19 vaccine acceptability [6]. The notable observation here pertained to the low level of vaccine confidence among young individuals. It might be attributed to their generally better health status, rendering them less susceptible to severe illness following a COVID-19 infection [29], thus potentially reducing their perception of the severity of infection. On the other hand, young individuals constituted a significant portion of the population actively engaged with social media, with many relying on these platforms as their primary source of health-related information [30]. Unfortunately, the internet also served as a breeding ground for vaccine-related misinformation, which could have adversely affected their confidence in vaccination [8,31]. Given young individuals’ extensive social interactions, they played a crucial role in virus transmission. Designing and leveraging social media and digital technologies, which enjoyed widespread popularity among young individuals, presented a potential avenue. For instance, a study by Lee et al [31] demonstrated the potential effectiveness of chatbot in enhancing vaccine confidence.

Comprehending the strength of conformity to social norms may provide insights into designing interventions. In countries with low culture tightness, there is a need to reinforce social norms linked to vaccination behaviors. Previous evidence has demonstrated the social norms could be altered (example of reducing drinking and driving in the USA) [32] and the country as a whole might tighten up on norms that have become too loose (example of reducing alcohol and drug abuse in Iceland) [33]. Health authorities should remain vigilant regarding vaccine-related misinformation and antivaccine sentiments on social media. Organizing town hall meetings or Q&A sessions can facilitate direct engagement between health practitioners and the public, fostering open dialogue and effectively addressing vaccine hesitancy.

In countries with high cultural tightness, engaging respected government officials or health experts to publicly endorse and promote vaccination may be effective. In such societies, authorities can frame vaccination as a civic duty and underscore the potential social repercussions associated with noncompliance. It should be noted that high level of cultural tightness, while conferring benefits, is not a panacea for the problem of vaccine hesitancy, and having a high-tightness culture does not guarantee a low risk of vaccine hesitancy. It is our supposition that, in high cultural tightness countries, low vaccination rates may be observed when there was limited awareness about vaccine availability and vaccination was not considered a social norm. This may be the case in China, which has the largest population in the world, yet only has a 1%‐3% influenza vaccination rate [34].

Our study has limitations. First, COVID-19 vaccine confidence was influenced by many unidentified factors (such as trust in authority), although we did our best to adjust numerous individual- and country- variables in the model. Second, our analysis was restricted to 28 countries for which cultural tightness and COVID-19 vaccine confidence data were available, which may limit the robustness of our results. Third, the association between cultural tightness and COVID-19 vaccine confidence should be examined on more nuanced levels (such across states within a country), particularly for large countries with diverse cultural structures like the United States. Fourth, while we adjusted for survey methodology by including it as a covariate in the regression models, the response biases caused by different data collection methods may still exist. Finally, given the diversity and complexity of religious beliefs in each country, we categorized them into three groups: “largest,” “minority,” and “refusal to answer,” potentially resulting in the loss of more nuanced findings in this domain.

### Conclusions

Our study revealed that countries with higher cultural tightness might be more likely to show greater COVID-19 vaccine confidence. Individuals’ sociodemographics, including younger age, female sex, and low educational level, were positively associated with low vaccine confidence. Our findings provided insights on designing tailor interventions to handle vaccine hesitancy.

## Supplementary material

10.2196/66872 Multimedia Appendix 1 Summary tables and figures on survey implementation, key constructs, country-level indicators, and model diagnostics.

## Abbreviations

GNI: gross national income
VCI: Vaccine Confidence Index
VCP: Vaccine Confidence Project
VIF: variance inflations factor
WHO: World Health Organization
