# Supplementary material for: The Association Between Cultural Tightness and COVID-19 Vaccine Confidence From 28 Countries: Cross-Sectional Study
Source: JMIR Public Health Surveill. 2025 Apr 24;11:e66872. doi: 10.2196/66872 (PMC12045521; doi:10.2196/66872)
Supplement: Multimedia Appendix 1 [file publichealth-v11-e66872-s001.docx]

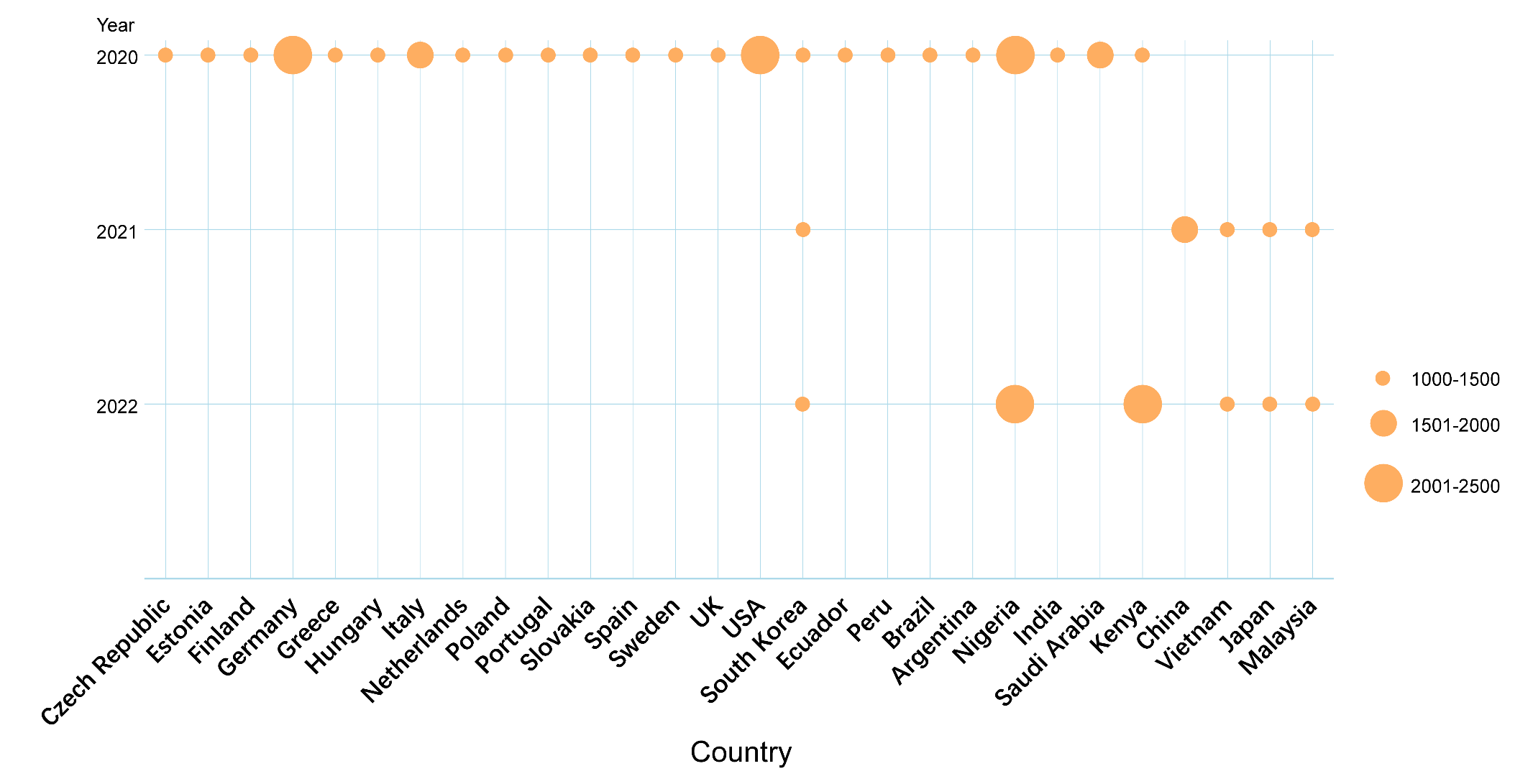


**Figure S1. Sample size by countries and years of surveys**

**Table S1. Survey fieldwork dates**

| **Country** | **Fieldwork dates** | **Methodology** |
| --- | --- | --- |
| Czech Republic | 13 March – 27 March, 2020 | Online |
| Estonia | 13 March – 27 March, 2020 | Online |
| Finland | 6 March – 3 April, 2020 | CATI & Face-to-face |
| Germany | 10-16 June, 10-16 June 2020 | Online |
| Greece | 13 March – 27 March, 2020 | Online |
| Hungary | 13 March – 27 March, 2020 | CATI |
| Italy | 13 March – 27 March, 10-16 June, 2020 | Online |
| Netherlands | 13 March – 27 March, 2020 | Online |
| Poland | 13 March – 27 March, 2020 | Online |
| Portugal | 13 March – 27 March, 2020 | Online |
| Slovakia | 13 March – 27 March, 2020 | Online |
| Spain | 13 March – 27 March, 2020 | Online |
| Sweden | 13 March – 27 March, 2020 | Online |
| UK | 5-13 June 2020 | Online |
| USA | 5-13 June 2020 | Online |
| South Korea | 15-18 June 2020; 5-14 July 2021; 19 May-1 June 2022 | Online |
| Ecuador | 16 June - 22 July, 2020 | CATI |
| Peru | 16 June - 22 July, 2020 | CATI |
| Brazil | 16 June - 23 July, 2020 | CATI |
| Argentina | 16-28 June 2020 | CATI |
| Nigeria | 15 - 28 June, 21 September-8 October, 2020; 19 January- 8 February, 2022 | Face-to-face |
| India | 23 June - 2 July, 2020 | CATI |
| Saudi Arabia | 25 June - 8 July, 2020 | CATI |
| Kenya | 08-24 October 2020; 14-24 January, 2022 | CATI & Face-to-face |
| China | June to August 2021 | Online |
| Vietnam | 16 June to 14 July 2021; 17-25 May 2022 | CATI |
| Japan | 5-8 July 2021; 17-26 May 2022 | Online |
| Malaysia | 14-27 June 2021; 20 May-20 June 2022 | Online |

CATI: computer-assisted telephone interview

**Table S2. Survey Items on cultural tightness–looseness**

| **Survey Items** |
| --- |
| There are many social norms that people are supposed to abide by in this country. |
| In this country, there are very clear expectations for how people should act in most situations. |
| People agree upon what behaviors are appropriate versus inappropriate in most situations this country. |
| People in this country have a great deal of freedom in deciding how they want to behave in most situations. |
| In this country, if someone acts in an inappropriate way, others will strongly disapprove. |
| People in this country almost always comply with social norms. |


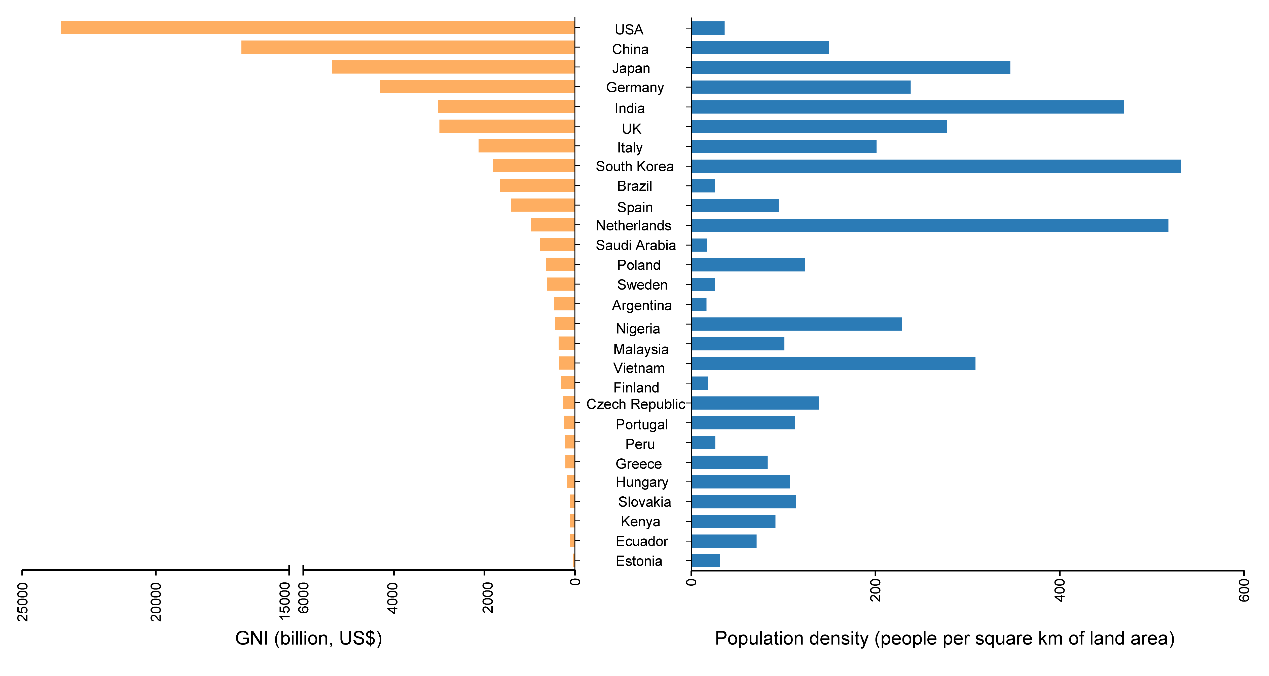


**Figure S2. GNI and population density by country**

**Table S3. Miss data in the sample#**

| **Variables** | **Number of missing variables** | **Percentage** |
| --- | --- | --- |
| Age group | 1 | 0.0% |
| Sex | 400 | 0.9% |
| Education level | 459 | 0.9% |

#Age group: Nigeria 1(0%); Sex: Germany 3 (0.15%), Greece 3 (0.3%), Hungary 1 (0.1%), Netherlands 1 (0.1%), Spain 4 (0.4%), Czech Rep 7 (0.7%), Estonia 7 (0.7%), Slovakia 5 (0.5%), Finland 7 (0.7%), Japan 90 (4.27%), South Korea 255 (7.76%); Education level: South Korea 262 (7.97%), Japan 136 (6.46%), Malaysia 29 (1.45%), Nigeria 2 (0.05%), Kenya 2 (0.06%).


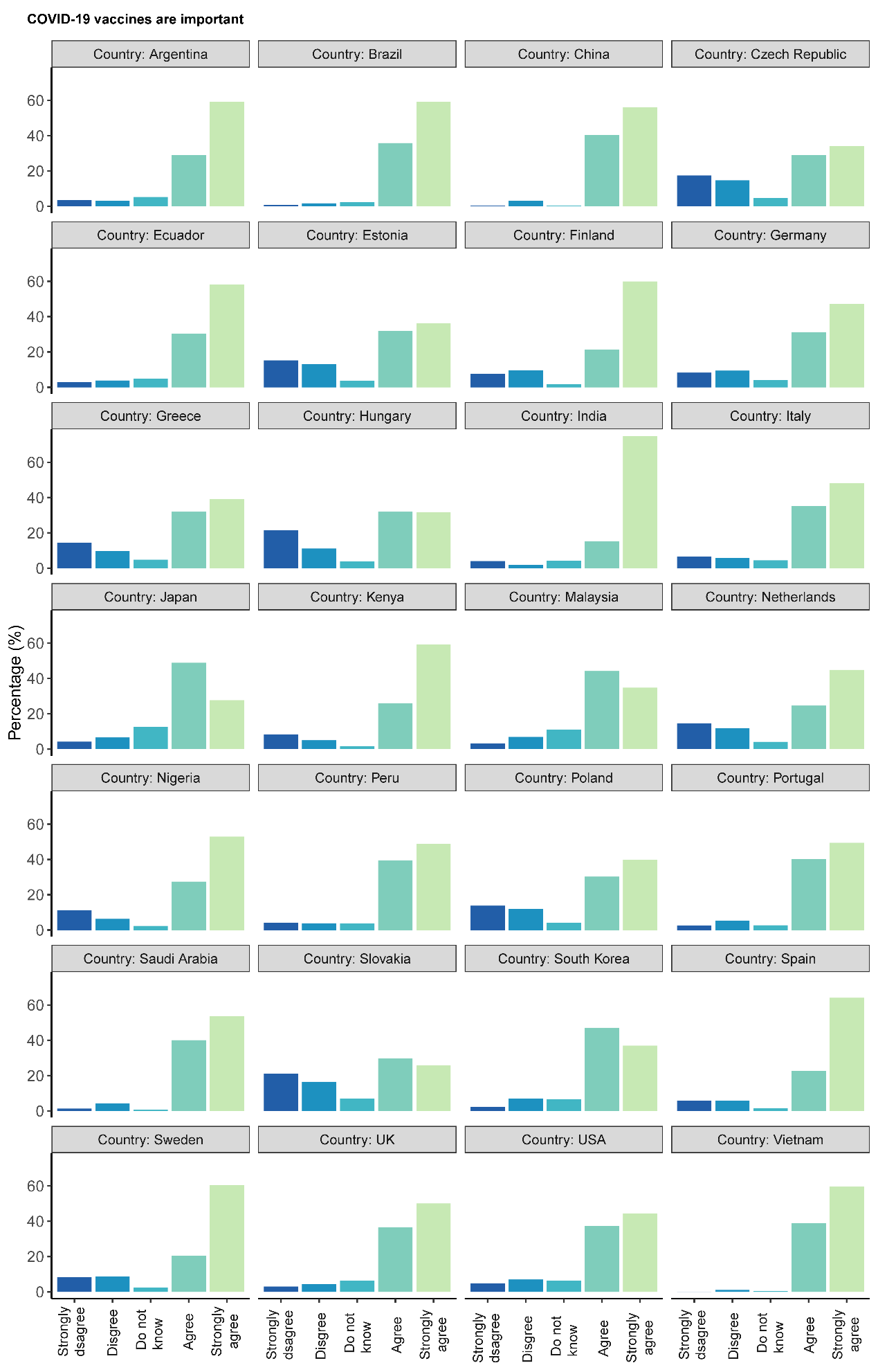


**Figure S3. Individual responses towards the importance of vaccine by country**


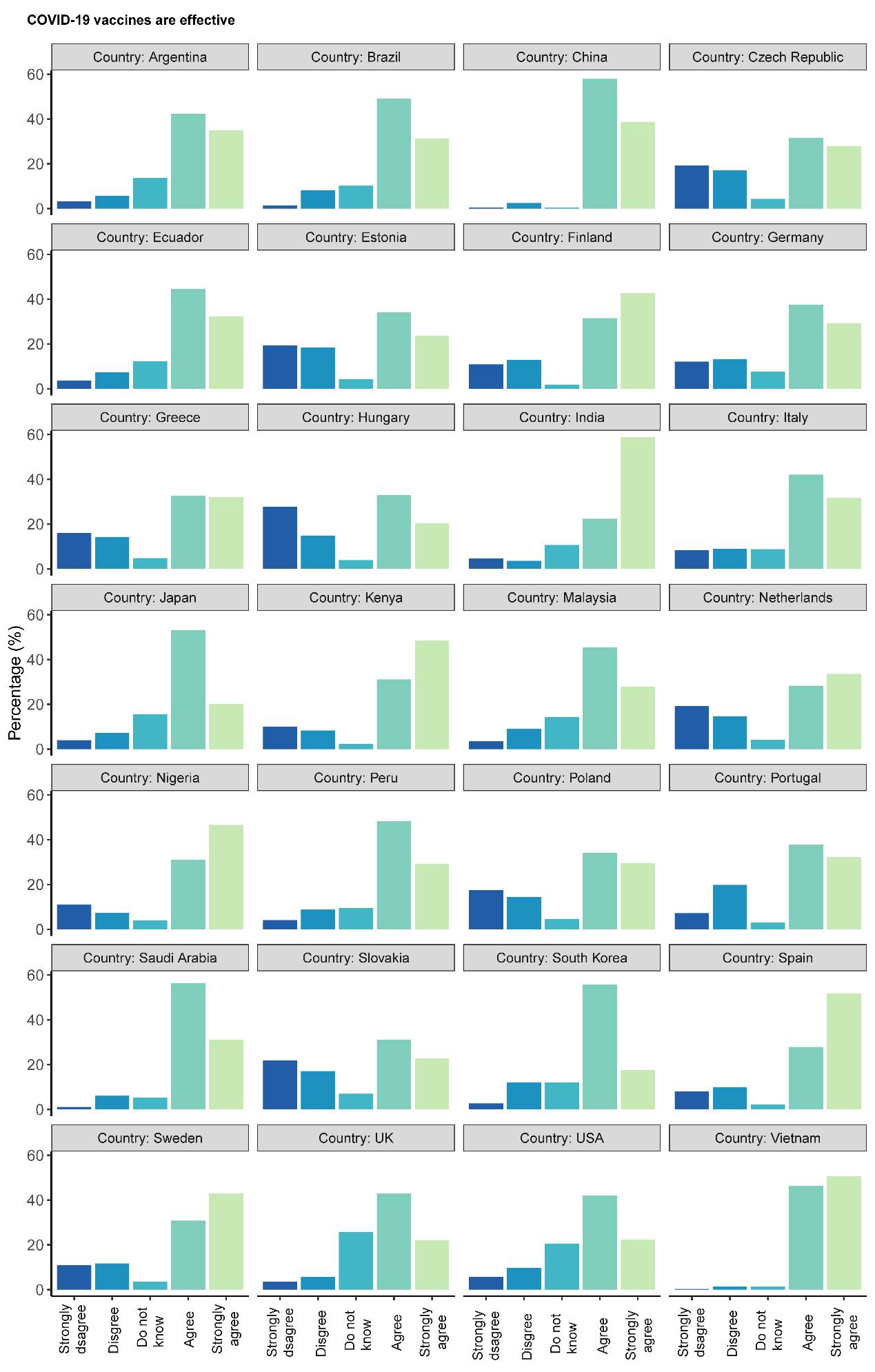


**Figure S4. Individual responses towards the effectiveness of vaccine by country**


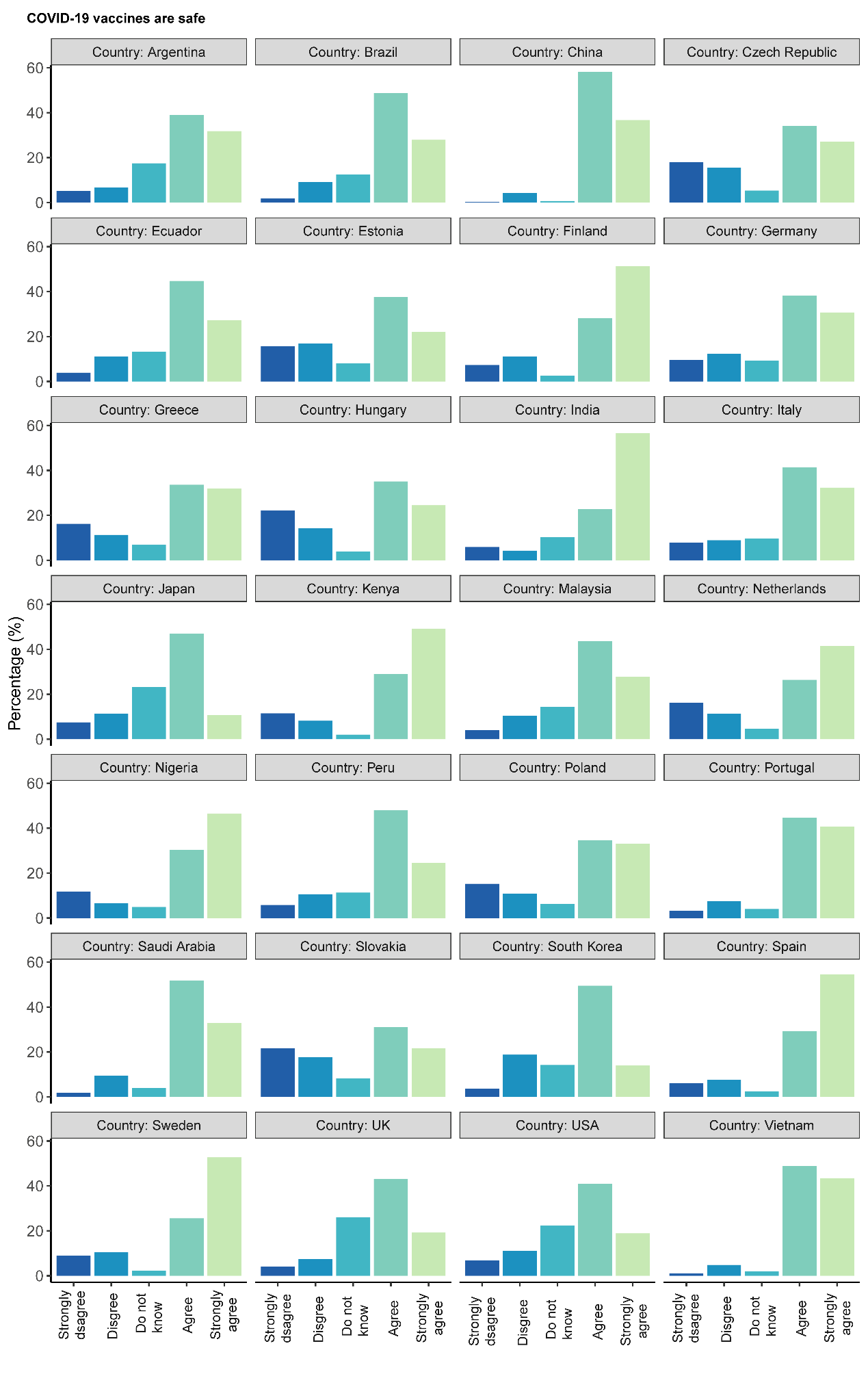


**Figure S5. Individual responses towards the safety of vaccine by country**

**Table S4. VIF for variables in the model 3**

| **Variables** | **Variance inflations factors** |
| --- | --- |
| Age | 1.052 |
| Sex | 1.004 |
| Education | 1.053 |
| Religious | 1.024 |
| Individual perceptions | 1.022 |
| Cultural tightness | 2.054 |
| GNI | 1.262 |
| Population density | 1.643 |
| Time | 1.451 |
| Survey methodology | 2.623 |
| Individualism | 2.473 |
| Power distance | 2.097 |
| Long-term orientation | 2.257 |
| Indulgence | 1.298 |
| Uncertainty avoidance | 1.600 |
| Individual perception × Cultural-tightness | 1.243 |
